# HMGB1 Promotes Prostate Cancer Development and Metastasis by Interacting with Brahma-Related Gene 1 and Activating the Akt Signaling Pathway

**DOI:** 10.7150/thno.33972

**Published:** 2019-07-09

**Authors:** Dao‑Jun Lv, Xian‑Lu Song, Bin Huang, Yu-Zhong Yu, Fang‑Peng Shu, Chong Wang, Hong Chen, Hai-Bo Zhang, Shan‑Chao Zhao

**Affiliations:** 1 Department of Urology, Nanfang Hospital, Southern Medical University/ The First School of Clinical Medicine, Southern Medical University, Guangzhou 510515, China.; 2 Department of Radiotherapy, Affiliated Cancer Hospital & Institute of Guangzhou Medical University, Guangzhou 510095, China.; 3 Department of Urology, Meizhou People's Hospital, No. 63, Huang Tang Road, Meizhou, 514031, Guangdong, People's Republic of China.

**Keywords:** * HMGB1*, epithelial-mesenchymal transition, Brahma-related gene 1, *Akt* pathway, prostate cancer

## Abstract

**Background and Aim**: We have previously shown that high-mobility group box 1 (*HMGB1*) is an independent biomarker for shortened survival of prostate cancer (PCa) patients. However, the specific role of *HMGB1* in tumor development and progression remains largely unknown. In this study, we investigated the molecular mechanisms of *HMGB1* in PCa tumorigenesis.

**Methods**: Gain-of-function and loss-of-function experiments were used to determine the biological functions of *HMGB1* both *in vitro* and *in vivo*. Bioinformatic analysis, immunoprecipitation, and immunofluorescence assays were applied to discern and examine the relationship between *HMGB1* and its potential targets. Specimens from 64 patients with PCa were analyzed for the expression of *HMGB1* and its relationship with Brahma-related gene 1 (*BRG1*) was examined by immunohistochemistry.

**Results**: The results demonstrated that ectopic expression of *HMGB1* facilitated growth and metastasis of PCa by enhancing *Akt* signaling pathway and promoting epithelial-mesenchymal transition (EMT), while silencing of *HMGB1* showed the opposite effects. Mechanistically, *HMGB1* exerted these functions through its interaction with *BRG1* which may augment *BRG1* function and activate the *Akt* signaling pathway thereby promoting EMT. Importantly, both *HMGB1* and *BRG1* expression was markedly increased in human PCa tissues.

**Conclusions**: Taken together, these findings indicate that upregulation of* HMGB1* promotes PCa development *via* activation of *Akt* and accelerates metastasis through regulating *BRG1*-mediated EMT. *HMGB1* could be used as a novel potential target for the treatment of PCa.

## Introduction

Prostate cancer (PCa) is the second most common human malignancy in men worldwide 1. Although the recent advances in diagnosis and surgical resection can cure a majority of patients with localized disease, nearly 20-30% of treated men inevitably progress to castration-resistant prostate cancer (CRPC) followed by metastasis 2, the leading cause of poor prognosis and mortality 3. However, the underlying molecular mechanisms in PCa metastasis remain unclear. Therefore, there is an urgent need to detect innovative diagnostic and therapeutic methods based on the biological and molecular mechanisms of metastatic spread of PCa.

High-mobility group box 1 (*HMGB1*), a member of HMG1-type polypeptides, was initially identified as a DNA chaperone that is involved not only in inflammation but also in cancer 4, 5. Aberrant expression and release of *HMGB1* are associated with various human carcinomas including colon, breast, lung, cervical, and liver cancers 6-10. During tumor progression and treatment, *HMGB1* has been shown to play different roles in facilitating both cell survival and death by modulating various signaling pathways, including inflammation, gene transcription, autophagy, metastasis, metabolism, and apoptosis 11. Several signaling pathways mediated by *HMGB1* have been implicated in carcinogenesis including *PI3K/Akt* signaling and epithelial-mesenchymal transition (EMT) process 12, 13. Our previous studies have revealed that *HMGB1* is up-regulated in human PCa 14. However, the precise role of intracellular *HMGB1* in metastasis during PCa tumorigenesis remains largely unknown.

In this study we explored the role of *HMGB1* in growth, invasion, and metastasis of PCa *in vitro* as well as tumorigenesis *in vivo*. Our results illustrated that *HMGB1* exerts these functions through constitutively activating the *PI3K/Akt* signaling pathway, modulating the expression of Brahma-related gene 1 (*BRG1*, also known as *SMARCA4*) in PCa cells, and regulating EMT via *BRG1*. Thus, our study has revealed a potential mechanism by which *HMGB1* mediates the development and progression of PCa.

## Materials & Methods

### Patients and clinical samples

Paraffin specimens were collected from radical prostatectomy (RP) performed at the Nanfang Hospital between 2012 and 2016; 14 benign prostatic hyperplasia (BPH) tissues were used as controls. Characteristics of patients were retrospectively obtained from the review of medical records. Among the selected patients, the median age was 69 years (range: 30-89 years). The clinical stages and Gleason Scores (GS) were reassessed based on the American Joint Committee on Cancer (AJCC) 2002 and the World Health Organization (WHO) classification system. The detailed clinicopathological data are shown in **Table S2**. All patients signed the informed consent to participate in the study according to the ethical protocols of the Ethics Committee of Nanfang Hospital, Southern Medical University.

### Cell lines and culture conditions

Human immortalized prostate epithelial cell line RWPE-1 and four PCa cell lines PC-3, DU145, 22Rv1, and LNCaP were purchased from Stem Cell Bank, Chinese Academy of Sciences. Cells were grown in RPMI1640 medium supplemented with 10% fetal bovine serum (FBS, Hyclone). RWPE-1 cells were cultured in Keratinocyte Serum Free Medium (KSFM) (Gibco, No. 10744-019) supplemented with 5 ng/mL epidermal growth factor (EGF) (Gibco, No. 10450-013), and 1% antibiotic-antimycotic solution (Gibco, No. 15140-122). All cell lines were maintained at 37 °C in 5% CO_2_.

### RNAi and gene transfection

Three small interfering RNAs (siRNAs) targeting *HMGB1* (si-*HMGB1*-1, 2, 3) and negative control (NC) siRNA with no specific target were synthesized by RiboBio Co. (Guangzhou, China). The siRNA sequences that specifically target HMGB1 are shown below: si-h-*HMGB1*_001: GTTGGTTCTAGCGCAGTTT; si-h-*HMGB1*_002:GGACAAGGCCCGTTATGAA; si-h-*HMGB1*_003: GAGGCCTCCTTCGGCCTTC.

Stable cell lines expressing *HMGB1* or sh-*HMGB1* were generated using the lentivirus vector (GeneChem Bio-Medical Biotechnology, Shanghai, People's Republic of China) according to the manufacturer's instruction. The sequence of short hairpin RNA (shRNA) against *HMGB1* was 5'-GGACAAGGCCCGTTATGAA-3'. Stable cell lines overexpressing *HMGB1* or sh-*HMGB1* were selected for 10 days with 5 μg/mL puromycin. The siRNA had previously been shown to knockdown *BRG1* expression efficiently and specifically 15.

### RNA extraction and Quantitative real-time PCR (qRT-PCR) assays

Total RNA was extracted from cells with RNAiso Plus reagent (TaKaRa) and cDNA was reverse- transcribed by using PrimeScript RT reagent Kit (TaKaRa). The RT-PCR analysis was conducted using the SYBR Green PCR Master Mix (TaKaRa) with Applied Bio-systems 7500 Fast Real-Time RCR System (Applied Biosystems, Foster City, CA, USA). Each measurement was performed in triplicate, and the results were normalized to the internal control of *GAPDH*. The relative expression of the target gene was determined by 2^-△△Ct^ method. The specific primers were as indicated: *HMGB1*: 5'-AAAGCGGACAAGGCCCGTTAT-3' (forward) and 5'-AAGAGGAAGAAGGCCGAAGGAG-3' (reverse). *BRG1*: 5'-CAGATCCGTCACAGGCAAAAT-3' (forward) and 5'-TCTCGATCCGCTCGTTCTCTT-3' (reverse). Primers set for *GAPDH* was 5'-CCAGGTGGTCTCCTCTGACTTC-3' (forward) and 5'-GTGGT CGTTGAGGGCAATG-3' (reverse).

### Cell viability

Cell viability was conducted using cell counting kit-8 (CK-04, Dojindo) according to the manufacturer's instructions. Briefly, PCa cells at 2 × 10^3^ per well were seeded into 96-well plates. Then, 10% v/v CCK-8 was added to each plate and the cells were incubated at 37 ℃ for 2 h. Subsequently, the optical density (OD) at 450nm at predetermined time points was measured by a microplate reader (EXL800, BioTek Instruments).

### Colony formation

PCa cells (300 cells/well) were seeded into six‐well plates and incubated for two weeks to allow colony formation. Then, the colonies were fixed with 4% paraformaldehyde and stained with Giemsa. Each group was replicated in three wells, and the colony formation experiment was performed with three replicates.

### 5-ethynyl-2'-deoxyuridine assay (EdU) incorporation Assay

Cell-Light ™ EdU staining kit (RiboBio, Guangzhou, China) was utilized to determine cell proliferation activity based on the manufacturer's instructions. Images were taken with a microscope (Olympus, Tokyo, Japan) at 200×. The proportion of EdU positively stained cells (with red fluorescence) to Hoechst-stained cells (with blue fluorescence) in per well was calculated.

### Cell-cycle analysis

Cell-cycle analysis was executed as described previously 16. In brief, human PCa cells were digested using 0.25% Trypsin/EDTA solution and fixed with ice-cold 70% ethanol at 4 ℃ overnight. Subsequently, cells were stained with 50 μg/mL propidium iodide (PI) (keygentec. Nanjing, China). DNA content of cells in each group was determined by flow cytometry (FACS Calibur, Becton Dickinson). Synchronization in the cell cycle analysis was not used; all assays were repeated for three times.

### Cell scratch assay

PCa cells were seeded at a density of 1 × 10^6^ cells/well in 6-well plates and grown to 75%-90%. Linear wounds were scratched with a 10 μL plastic pipette tip and the cell debris was washed away with PBS twice. Subsequently, cells were incubated in FBS-free culture medium. Wounds of the scraped area were monitored and captured at indicated time points with an inverted microscope (Olympus IX71) at 100× magnification. Distance from each side of the scratch was quantified at 3 distinctive fields within the same scratch.

### Transwell migration assay

Cell migration assay was conducted utilizing Transwell inserts (Costar, Corning, Cambridge, MA, USA) with a chamber (8.0μm pore size). 5 × 10^4^ cells were resuspended in serum-free medium and seeded into the upper compartment of the insert. Then, 500μL complete medium was added to the lower chamber. After 36 h, the cells were fixed with 4% paraformaldehyde and stained with Giemsa (Boster Ltd., Wuhan, China). Subsequently, cells on the top surface of the membrane were removed, and those on the bottom surface were photographed using an inverted microscope (Olympus DP72) at 200× magnification. Five randomly chosen visual fields were captured and counted using Image J software. The number of cells migrating through the chambers in each group was documented as the average value.

### Transwell invasion assay

For invasion assay, the Transwell membrane was pre-coated with Matrigel matrix (40111ES08; Yisheng Biotechnology Co., Ltd., Shanghai, China). Subsequently, 6 × 10^4^ PCa cells were seeded in the upper section of the Boyden chambers. After 36 h of culture at standard conditions, the superficial cell layer was wiped off with cotton balls before visualization. The next steps were the same as in the migration assay.

### Mice xenograft and tumor metastasis

All experimental animal procedures were authorized by the Animal Care and Use Committee of Southern Medical University and animals were raised under Specific Pathogen Free (SPF) conditions. Xenograft tumor models were generated by subcutaneous injection of 2×10^6^ PC3/sh-Ctrl, PC3/sh- *HMGB1*, 22Rv1/vector, and 22Rv1/Lv-*HMGB1* cells (n = 6 per group), on the axillae of 4 to 6 weeks BALB/c nude mice obtained from the Animal Center of Southern Medical University, Guangzhou, China. Tumor size was measured every 5 days and the volume was calculated using the formula: volume = (length × width^2^)/2 16. To assess metastasis, 5 × 10^6^ cells in 100μL of PBS were injected via the tail veins of mice (n = 6 for each group). After 40 days, lung metastases of tumor nodules were observed under the microscope. Image J software was used to calculate the lung colonization of foci in three randomly selected fields. The presence of cancer cells in the tumor tissues was verified with H&E (hematoxylin and eosin) staining and immunohistochemistry (IHC) staining using antibodies against *HMGB1* and *Ki-67* (Abcam, #16667).

### Western blot analysis

Cells were extracted using RIPA lysis buffer containing protease inhibitors (#KGP250, KeyGEN BioTECH, Nanjing, China) according to the manufacturer's protocol. After extraction, equal amounts of proteins in the cell lysates were separated by SDS/PAGE gels (4-12%, Bio-Rad) and electrically transferred onto polyvinylidene fluoride (PVDF, Millipore) membranes. Subsequently, membranes were blocked with 5% no fat milk or bovine serum albumin (BSA) and incubated for 16 h at 4 ℃ with the following specific primary antibodies: rabbit anti- *HMGB1* (#ab18256, Abcam) and rabbit anti-*BRG1* (#ab110641, Abcam); EMT marker (#9782, Cell signaling, MA, USA), rabbit anti-*cyclin D1* (#2978), rabbit anti-*c-myc* (#5605), rabbit anti-*p27 Kip1*(#3686), rabbit anti-*p21 Waf1/Cip1*(#2947), rabbit anti-*CDK4* (#12790), rabbit anti-*Akt* (#4691), rabbit anti-*phospho- Akt^Ser473^*(#4060) and rabbit anti-*phospho-Akt^Thr308^*(#13038). These antibodies were purchased from Cell Signaling Technology except for mouse anti-*β-actin* (#60008-1-Ig, Proteintech Group) and rabbit anti-*α- tubulin* (#ab18251, Abcam). Subsequently, all membranes were immersed in horseradish peroxidase- linked secondary anti-rabbit IgG or anti-mouse IgG antibodies (Cell Signaling Technology) for 1 h at room temperature. The bands were visualized using the enhanced chemiluminescence (ECL) detection system (Pierce Biotechnology, Rockford, IL, USA). The intensity of protein bands was quantified with the Image J software.

### Immunofluorescence assays

Cells were fixed with 4% paraformaldehyde (E672002, Sangon Biotech) for 15 min at room temperature followed by incubation with 0.5% Triton solution (A110694, Sangon Biotech) for 10 min to permeabilize the cell membrane. Then, cells were incubated with Tris-buffered saline containing 5% bovine serum albumin (BSA) for 30 min. Subsequently, samples were incubated with rabbit anti-*HMGB1* (#ab18256, Abcam) and mouse anti- *BRG1* (#sc17796, Santa Cruz) at 4 ℃ overnight. Finally, the fluorescent secondary antibody Alexa Fluor 488-conjugated goat anti-rabbit IgG (#4412S, Cell Signaling Technology) and Alexa Fluor 594-conjugated goat anti-mouse IgG (#8890, Cell Signaling Technology) were used to detect primary antibodies. DAPI (E607303, Sangon Biotech) was applied for nuclear staining. Fluorescence images were visualized and collected under inverted confocal microscopy (DM5000B, Leica).

### Plasmid transfection

The full-length human wild-type *Akt* and *BRG1* cDNAs were inserted into PENTER-3FLAG-SV40- Kana expression vector obtained from Shangdong Vigene Biosciences Co. All constructs were validated by sequencing. PC-3/sh-*HMGB1* and LNCaP/sh- *HMGB1* cells were transiently transfected with pUSEamp-myr-akt or pUSEamp empty vector and *BRG1* or empty vector by using Lipofectamine™ 3000 Transfection Reagent following the manufacturer's instructions (L3000015, Thermo Fisher Scientific, USA). Plasmid expression was verified by Western blotting.

### Co-immunoprecipitation

Co-immunoprecipitation of proteins was executed as previously described 17 with modifications. In brief, PC-3 and LNCaP cell protein supernatants were pre-treated with 50 μL A/G beads (Selleck Chemicals, Houston, U.S.A) before immunoprecipitation and then with 5 μg control IgG (Santa Cruz Biotechnology), *HMGB1*, or *BRG1* antibodies overnight at 4 °C. After further incubation with 50 μL A/G beads at 4 °C for 6 h, the immune-precipitates were eluted with ice-cold PBS containing 0.2% NP-40 for 5 times. Subsequently, these immunoprecipitated proteins were electrophoresed on SDS-PAGE, and visualized by silver staining (Byeotime, Shanghai, China). LC-MS/MS 18 was used to analyze the digested gels. The immunoprecipitated protein complexes were subsequently isolated by metal boiling in 2 × SDS-PAGE sample buffer for 10 min and utilized for immunoblotting with both anti-*HMGB1* (#ab18256, Abcam) and anti-*BRG1* (#ab110641, Abcam) antibodies.

### Immunohistochemical analysis and evaluation

Tissue microarray (TMA) and IHC were employed to investigate protein expression in tissues as described previously 16, 19, 20. For IHC, the specimens were incubated with antibodies against *HMGB1* (1:500), and *BRG1* (1:50). Positive cells were scored as follows: 0 (no staining, 0%), 1 (staining range, 1-25%), 2 (staining range, 26-50%), 3 (staining range, 51-75%), or 4 (staining range, 76-100%). Intensity scores were recorded as: 0 (no staining), 1 (weakly staining, light yellow), 2 (moderately staining, yellowish brown), and 3 (strongly staining, brown). The multiplication of the above two scores was recorded as follows: - (0-1), +(2-4), ++ (5-8), +++ (9-12). A score of ≥2 was regarded as upregulated, while <2 score was considered as low expression. In case of discrepancies (>5%), the results were reevaluated. All evaluations were performed by three independent senior pathologists using the same microscope.

### Statistical analysis

All statistical analyses were conducted using SPSS version 19.0 software (SPSS, Chicago, IL, USA). Numerical data were expressed as means ± SD. Differences between variables were analyzed by two-tailed Student t-test or One-way analysis of variance (ANOVA) for continuous variable groups. When ANOVA was significant, post hoc testing of differences between groups was carried out using the LSD test. Kaplan-Meier survival curve was plotted from the mice survival data and examined by the log-rank test. Correlations between the protein abundance and clinicopathological factors in PCa tumor tissues and BPH tissues were confirmed by *Pearson's* chi-square test or Fisher exact test for categorical/binary measures. *HMGB1* and *BRG1* expressions were explored by Spearman's correlation. *P*<0.05 was considered as statistically significant.

## Results

### HMGB1 accelerates PCa cell proliferation *in vitro*

**Figure 1:**
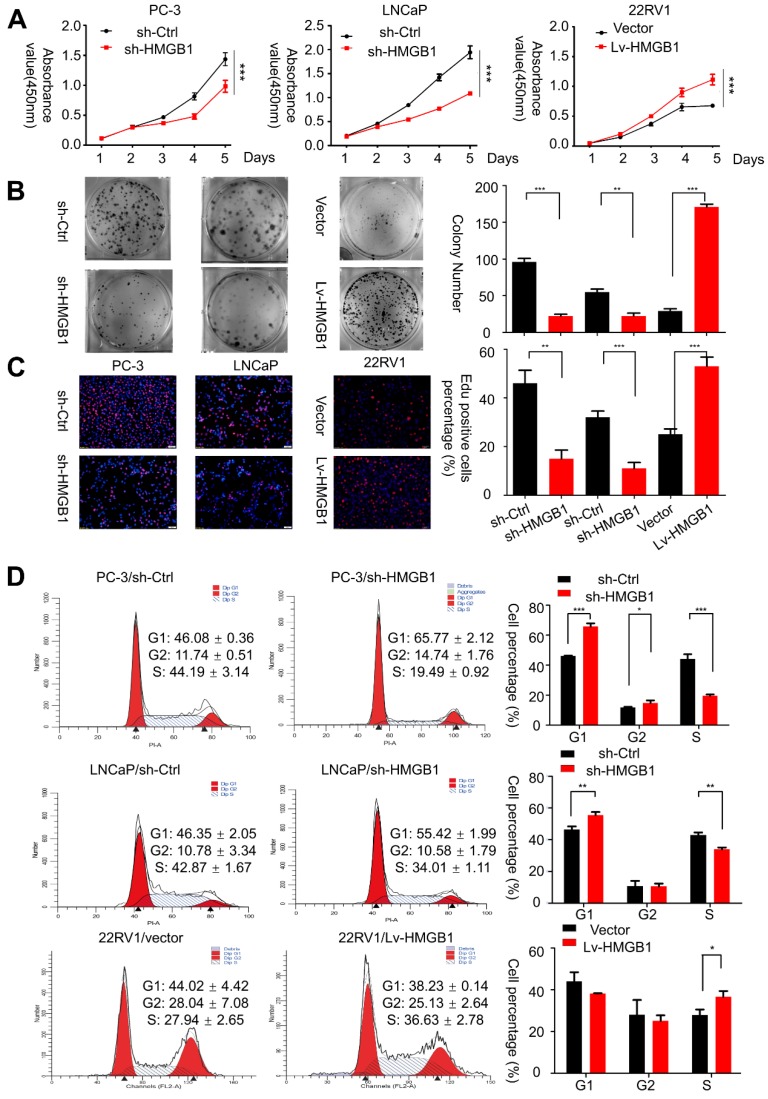
***HMGB1* promoted the aggressive behavior and proliferation ability of PCa cells *in vitro*.** (**A** and** B**) CCK-8 proliferation assay and colony-formation assay were used for detecting the proliferation ability in *HMGB1*-knockdown or -overexpressing PCa cell lines. (**C**) Representative micrographs (left) and quantification (right) of EdU incorporation. (**D**) Cell-cycle analysis revealed that knocking down *HMGB1* expression in PC-3 and LNCaP cells increased the percentage of cells in the G_1_ phase and decreased the percentage in S phase, while ectopic expression of *HMGB1* decreased the percentage of cells in the G_1_ phase and increased the percentage of cells in S phase, ^*^P<0.05, ^**^P<0.01, ^***^P<0.001.

To verify whether *HMGB1* is essential for PCa oncogenesis, we first analyzed the endogenous protein expression of HMGB1 in four tumor-derived PCa cell lines (PC-3, DU145, 22Rv1, and LNCaP) by Western blotting. PC-3 and LNCaP cells exhibited relatively higher levels of *HMGB1* protein than other cells, while 22Rv1 cells expressed *HMGB1 at* a much lower level (**Figure S1A**). Hence, we chose PC-3, LNCaP, and 22Rv1 cells for the subsequent studies. First, three siRNAs targeting *HMGB1* were designed to silence its expression in PC-3 and LNCaP cell lines. We chose si-*HMGB1*#2 for the subsequent experiments due to the highest inhibitory efficiency (**Figure S1B**). Then, we established stable silenced *HMGB1* in PC-3 and LNCaP cells as well as ectopic *HMGB1* in 22Rv1 cells by using lentiviral vectors. Western blot and RT-PCR analyses revealed that sh-*HMGB1* significantly silenced intracellular *HMGB1* protein compared with the control (sh-Ctrl), while the *HMGB1* overexpression group showed a dramatically increased level of *HMGB1* protein (**Figure S1C**). CCK-8 assay demonstrated that the proliferation of *HMGB1*-silenced cells was remarkably decreased but was increased in ectopic-*HMGB1*-expressing cells (**Figure 1A**). Consistent with this, the colony-forming ability was notably suppressed in PC-3/sh-*HMGB1* and LNCaP/sh-*HMGB1* cells compared with sh-Ctrl cells but was dramatically increased in 22Rv1/Lv-*HMGB1* cells (**Figure 1B**).

Then immunofluorescent staining for EdU incorporation assays showed increased DNA synthesis in 22Rv1 cells with ectopic expression of *HMGB1*, whereas blocking of *HMGB1* significantly suppressed DNA synthesis (**Figure 1C**). Additionally, flow cytometry analysis was conducted to confirm whether the change of proliferation is attributed to alterations in the cell cycle profile. Results showed that *HMGB1* knockdown in PC-3 and LNCaP cells induced G_1_ arrest while ectopic expression of *HMGB1* in 22Rv1 cells accelerated cell cycle progression into the S phase (**Figure 1D**). These results indicated that *HMGB1* enhances cell growth at least partially by inducing G_1_/S transition in PCa cells.

### HMGB1 facilitates PCa cell invasion and migration *in vitro*

**Figure 2:**
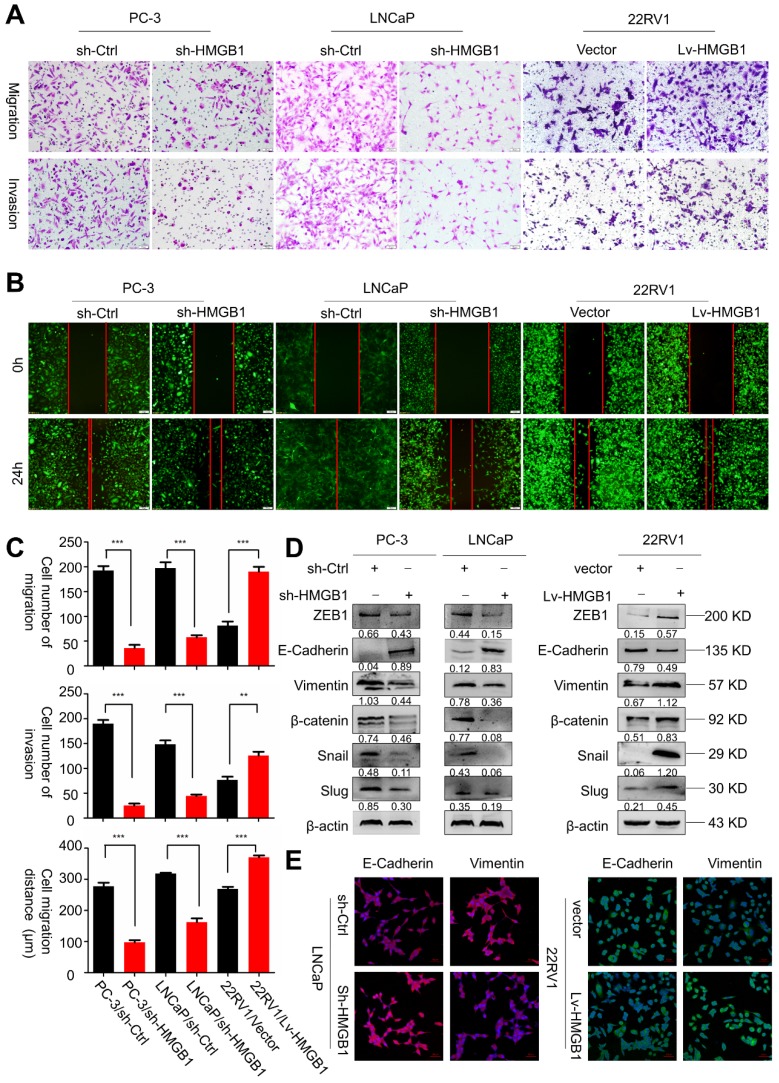
***HMGB1* facilitated migration and invasion of PCa cells *in vitro*.** (**A-B**) *HMGB1* knockdown markedly attenuated cell migration and invasion in PC-3 and LNCaP cells, while up-regulation of *HMGB1* increased the migration and invasion ability of 22Rv1 cells as measured by Transwell migration and wound healing assays. The invasive capability was determined by using Matrigel invasion chambers. (**C**) Graphical illustration of statistical results of transfection of *HMGB1*-shRNA or ectopic-*HMGB1* on cell invasion and migration. Data are presented as mean ± SD (n = 3). Migrated cells were counted using Image J software and represent mean values per field from at least three fields. Experiments were carried in duplicate. *P<0.05; **P<0.01; ***P<0.001 compared with the control (**D**) EMT markers were detected by Western blotting in both PCa cell lines with *HMGB1* knocked down or overexpressed. *β-actin* served as the loading control. (**E**) Immunostaining of mesenchymal and epithelial markers in LNCaP/sh-Ctrl and LNCaP/sh-*HMGB1* or 22Rv1/vector and 22Rv1/Lv-*HMGB1* cells as indicated, Scale bar = 50 μm.

Next, we sought to determine the role of *HMGB1* in tumor invasion and metastasis. Results of migration and invasion *in vitro* assays showed that depleted *HMGB1* suppressed cell migration and invasion of PC-3 and LNCaP cells (**Figure 2A-B**). Opposite effects on cell migration and invasion were observed in ectopic-*HMGB1*-expressing 22Rv1 cells (**Figure 2C**). Since EMT has been widely regarded as a crucial process in tumor invasion and metastasis 21, we hypothesized that *HMGB1* affected PCa cell migration and invasion through regulating the EMT progression. Western blotting and immunofluorescence analyses were performed to detect the protein expression of EMT-related markers. As anticipated, results demonstrated that *HMGB1* depletion reduced the abundance of mesenchymal markers *vimentin*, *β-catenin*, *Snail*, *Slug,* and *ZEB1*, but increased protein abundance of epithelial marker *E-cadherin* in PC-3 and LNCaP cells. In contrast, overexpression of *HMGB1* displayed the opposite effect (**Figure 2D-E**). These results suggested that *HMGB1* promotes invasion and tumor metastasis of PCa cells by regulating EMT.

### HMGB1 promotes the growth and metastasis of human PCa cells *in vivo*

**Figure 3:**
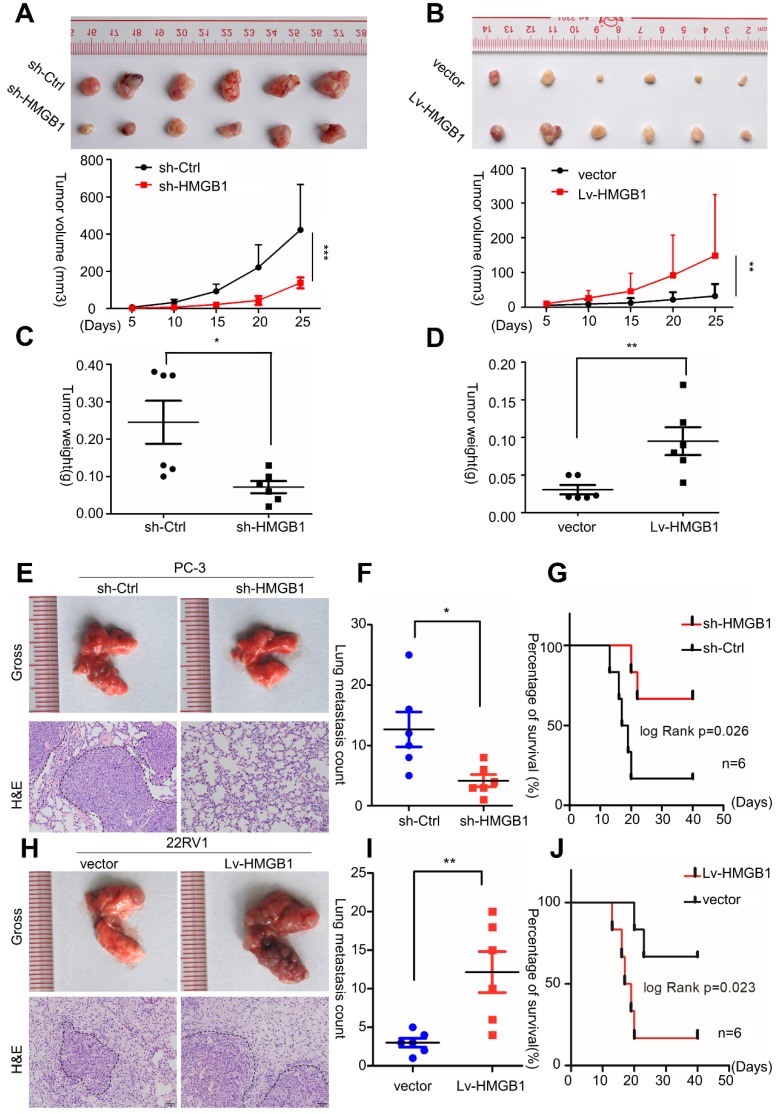
***HMGB1* enhanced tumor growth and metastasis *in vivo*.** Cells were injected into the hindlimbs of nude mice (n = 6). (**A** and** B**) Representative images of the gross tumors are shown (upper panels), Tumor growth curves were measured during the growth of the tumors (lower panels). Tumors derived from PC-3 cells expressing sh-*HMGB1* grew significantly slower than those from cells with sh-Ctrl, whereas ectopic expression of *HMGB1* in 22Rv1 cells dramatically enhanced tumor growth. (**C** and** D**) Final tumor weights were measured in each group. (**E** and** H**) Gross and microscopic illustrations of lung metastases in mice injected with PC-3/sh-Ctrl and PC-3/sh-*HMGB1* cells or 22Rv1/Vector and 22Rv1/Lv-*HMGB1* cells (n = 6). The lung sections were stained with H&E (200×). (**F** and** I**) The number of metastatic nodules in individual mice was counted under the microscope, ^*^P<0.05, ^**^P<0.01. (**G** and** J**) Kaplan-Meier overall survival curves for mice with PCa stratified by *HMGB1* knockdown and negative control (log-rank test, n = 6, *P* = 0.026), as well as overexpressed *HMGB1*and negative control (log-rank test, n = 6, *P* = 0.023), respectively.

To validate the potential impact of *HMGB1* depletion on PCa cell proliferation* in vivo*, PC-3/sh*-HMGB1* cells and PC-3/sh-Ctrl cells were injected subcutaneously in nude mice. Tumors in mice implanted in PC-3/sh-HMGB1 cells grew slower in comparison with control cells. *HMGB1* knockdown cells exhibited significantly smaller tumor volume and weight than control cells 25 days after injection (**Figure 3A-C**). The 22Rv1 cells with *HMGB1* overexpression, on the other hand, showed rapid growth speed than the control vector cells (**Figure 3B-D**). H&E staining showed the histopathological features of the tumor tissues. The expression of *Ki-67* proliferation antigen was dramatically weaker in tumor tissues with sh-*HMGB1* cells and stronger in xenografts with *HMGB1* overexpression as seen by IHC staining (**Figure S2**). These results provided evidence that *HMGB1* is a remarkable determinant for PCa cell growth.

To investigate the effects of *HMGB1* on PCa metastasis *in vivo,* tail vein xenograft model was generated. The tumor presence was validated by histological examination (**Figure 3E**-**H**). Results demonstrated that mice injected with *HMGB1* shRNA cells produced less lung colonization and peritoneal colonization compared to those with the control cells (**Figure 3E-F**). Moreover, we found that knockdown of *HMGB1* could prolong the survival time of mice compared with the control group (**Figure 3G**) while overexpression of *HMGB1* exhibited the reverse effects (**Figure 3H-J**). These findings corroborated our previous data 14 and demonstrated that *HMGB1* has a key role in PCa cell growth and metastasis *in vivo*.

### HMGB1 promotes PCa cell growth and proliferation through the PI3K/Akt pathway

**Figure 4:**
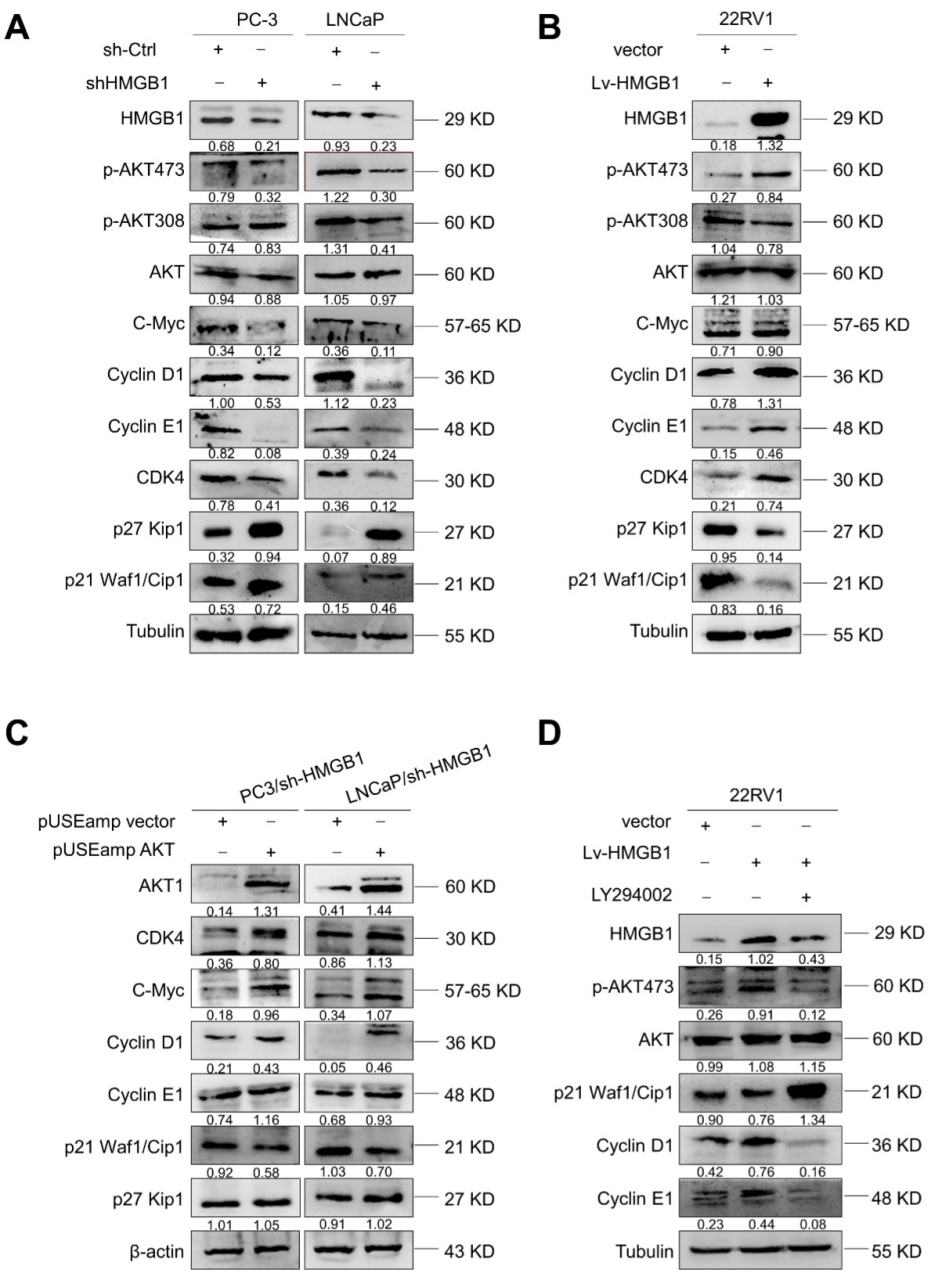
***HMGB1* activated Akt signaling pathway and increased key cell-cycle regulatory proteins.** (**A**) Western blotting revealed that *HMGB1* knockdown in PC-3 and LNCaP cells caused a decrease in *p-Akt ^Ser473^*, *CDK4*, *c-myc*, *cyclin D1*, and *cyclin E*, along with an increase in *p27*, *p21*. (**B**) Expression of these proteins showed an opposite effect in 22Rv1 cells with ectopic-*HMGB1*. (**C**) Introduction of *Akt* into PC-3 and LNCaP *HMGB1*-shRNA cells restored the levels of *CDK4*, *c-myc*, *cyclin D1*, and *cyclin E* together with decreased expression of *p21*; the *p27* level showed less change. (**D**) 22Rv1/Lv-*HMGB1* cells were treated with *Akt* inhibitor LY294002 (50 μM) for 24 h. Inhibition of the *Akt* signaling suppressed the promoting effect of *HMGB1* overexpression on the *Akt* pathway.

Since *HMGB1* promoted G_1_/S transition of PCa cells, we further investigated the key cell-cycle regulation signaling networks. Indeed, we observed an increase in *p21 Waf1/Clip1* and *p27 Kip1* levels and a decrease in phosphorylated *Akt^Ser-473^*, *CDK4*, *C-myc*, *cyclin D1*, and *cyclin E* levels with *HMGB1* deletion in PC-3 and LNCaP cells **(Figure 4A)**. Conversely, overexpression of *HMGB1* exhibited the opposite effects (**Figure 4B**). To further address whether the *HMGB1* was required for *Akt*-mediated promotion of PCa proliferation, we transiently transfected active Akt1 into PC-3/sh-*HMGB1* and LNCaP/sh-*HMGB1* cells and measured the proliferation. CCK-8 and colony formation assays showed that the *HMGB1*-depleted cells regained high proliferative ability upon upregulation of *Akt* (**Figure S3A-B**). Also, the ratio of cells in the G_1_ phase reduced concurrently with a high proportion in S phase (**Figure S3C**). Subsequently, Western blotting was performed to evaluate the effect of myc-*Akt* overexpression on the levels of cell-cycle-related proteins in PC-3-sh*-HMGB1* and LNCaP-sh*-HMGB1* cells. The up-regulated expression of *Akt* in *HMGB1***-**depleted cells increased the expression of *CDK4*, *c-myc*, *cyclin D1,* and *cyclin E*, together with decreased expression of *p21 Waf1/Clip1*, whereas the level of *p27 Kip1* was not altered (**Figure 4C**). These data indicated the existence of an underlying mechanism, other than the *Akt* pathway, in *HMGB1*-mediated downregulation of *p27 Kip1* expression. Our results also demonstrated that treatment with *PI3K* inhibitor LY294002 partially restored *p21 Waf1/Clip1* expression and decreased *p-Akt^Ser-473^*, *cyclin D1*, and *cyclin E* levels in 22Rv1 cells with ectopic expression of *HMGB1* (**Figure 4D**). Functional assays also demonstrated that inhibition of *Akt* signaling blocked the *HMGB1*-mediated up-regulation of PCa cell proliferation and cell cycle progression (**Figure S3D-F**). Taken together, these results further indicated that *Akt* pathway might involve in *HMGB1*-induced PCa cell proliferation.

### HMGB1-mediated EMT in PCa cells is regulated by BRG1-induced activation of the Akt signaling pathway

**Figure 5:**
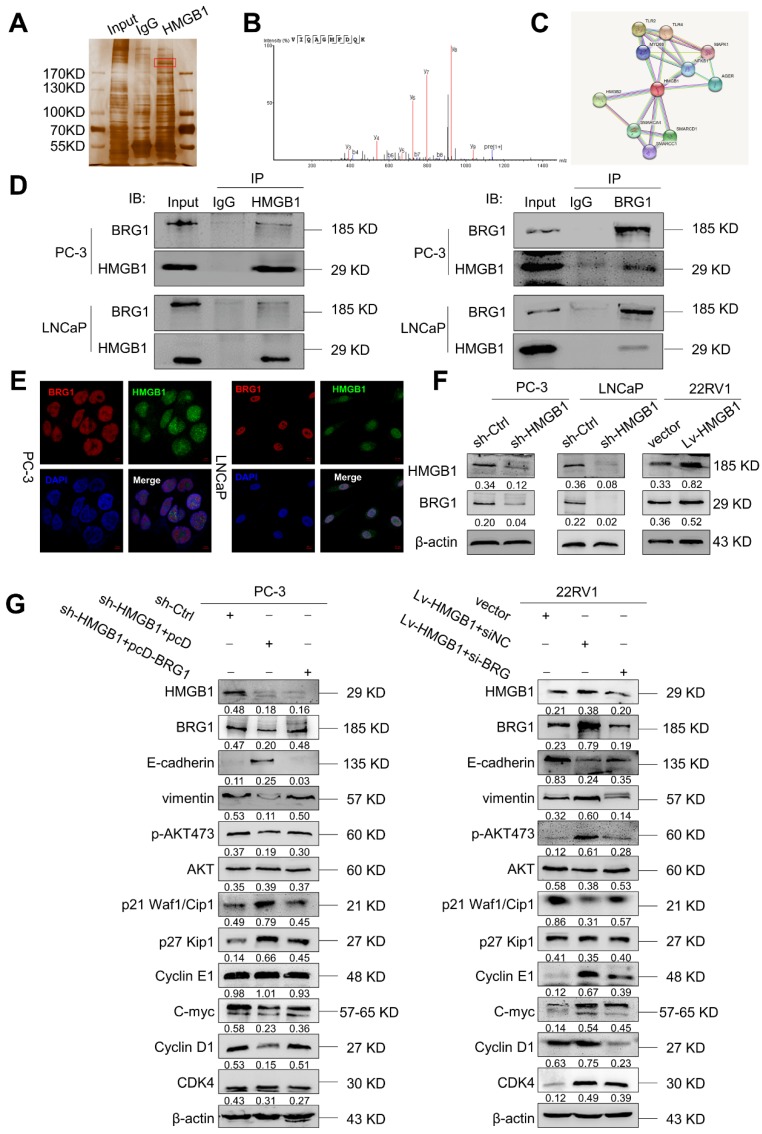
***BRG1* is an interactive factor of* HMGB1*.** (**A**) Immunoprecipitation and silver staining were performed by using PC-3 cell lysate with the anti-*HMGB1* antibody. (**B**) The spectrogram of the differential protein band was identified as *SMARCA4* also known as *BRG1* (Quadrupole Mass Spectrometer). (**C**) The single protein function partner network of *HMGB1* in String analysis. (**D**) Coimmunoprecipitation was performed to validate the interaction between *HMGB1* and *BRG1* in PC-3 and LNCaP cells. (**E**) Confocal immunofluorescence analysis showed the presence and localization of *HMGB1* and *BRG1* in the nuclei of PC-3 and LNCaP cells, Scale bar = 10 μm. (**F**) *HMGB1* regulated *BRG1* protein expression in PCa cells. Knockdown of *HMGB1* attenuated *BRG1* protein expression in PC-3 and LNCaP cells, whereas ectopic expression of *HMGB1* facilitated *BRG1* protein expression in 22Rv1 cells. (**G**) *HMGB1* mediated *Akt* pathway and EMT *via BRG1* in PCa cells. *BRG1* overexpression in *HMGB1* deficient PC-3 cells rescued the phosphorylation of *Akt* and EMT, while knockdown of *BRG1* in ectopic *HMGB1*-overexpressing 22Rv1 cells attenuated these effects.

To elucidate the potential molecular mechanism of *HMGB1* in PCa cells, we immunoprecipitated the *HMGB1* protein with an anti-*HMGB1* antibody and identified the proteins that may directly interact with *HMGB1* by LC-MS/MS. Among the *HMGB1*- interacting proteins identified by mass spectrometry (**Figure 5A-B, Table S1**), *BRG1* had been reported to drive the progression of PCa 22, 23. In addition, the single protein function partner network of *HMGB1* in String analysis also indicated that *BRG1* may interact with *HMGB1* (**Figure 5C**). To validate the protein- protein interaction between *HMGB1* and *BRG1*, co-immunoprecipitation (Co-IP) with an antibody against *HMGB1* was carried out. After immunoprecipitation with *HMGB1* conjugated beads, *BRG1* was found in the lysates from both PC-3 and LNCaP cells (**Figure 5D**). It has been reported that *ZEB1/BRG1* transcriptionally regulates *E-cadherin* expression and EMT that is implicated in the initial stages of tumor invasion 24. Thus, we hypothesized that *HMGB1* may interact with *BRG1* protein to induce EMT. As expected, in a reciprocal Co-IP with *BRG1* conjugated beads, *HMGB1* precipitated with *BRG1* (**Figure 5D**). Moreover, *HMGB1* and *BRG1* co-localized in both cell lines in the cell nucleus as observed by immunofluorescent staining (**Figure 5E**) indicating the physical interaction between *HMGB1* and *BRG1* in PC-3 and LNCaP cells. To confirm whether *HMGB1* regulates *BRG1* expression, immunoblotting was conducted to detect the abundance of *BRG1* in PCa cells when *HMGB1* expression was altered. Notably, *BRG1* expression was downregulated in PC-3 and LNCaP cells with *HMGB1* knockdown, while the level of *BRG1* was dramatically upregulated in 22Rv1 cells with *HMGB1* overexpression (**Figure 5F**). To validate the role of the *HMGB1-BRG1* axis in *HMGB1*-mediated EMT, we examined whether *BRG1* overexpression and silencing would reverse the effects of *HMGB1* knockdown and ectopic expression in PCa cells. When we induced *BRG1* siRNA in *HMGB1* overexpressing cells, immunoblotting showed reversed EMT markers and inhibition of phosphorylation of *Akt^ser473^* accompanied by suppression of cell cycle-related proteins (**Figure 5G**). These data demonstrated that *HMGB1* triggers EMT in PCa partly by enhancing *BRG1* activity.

### HMGB1-mediated EMT in PCa cells is regulated by BRG1-induced activation of the Akt signaling pathway

**Figure 6:**
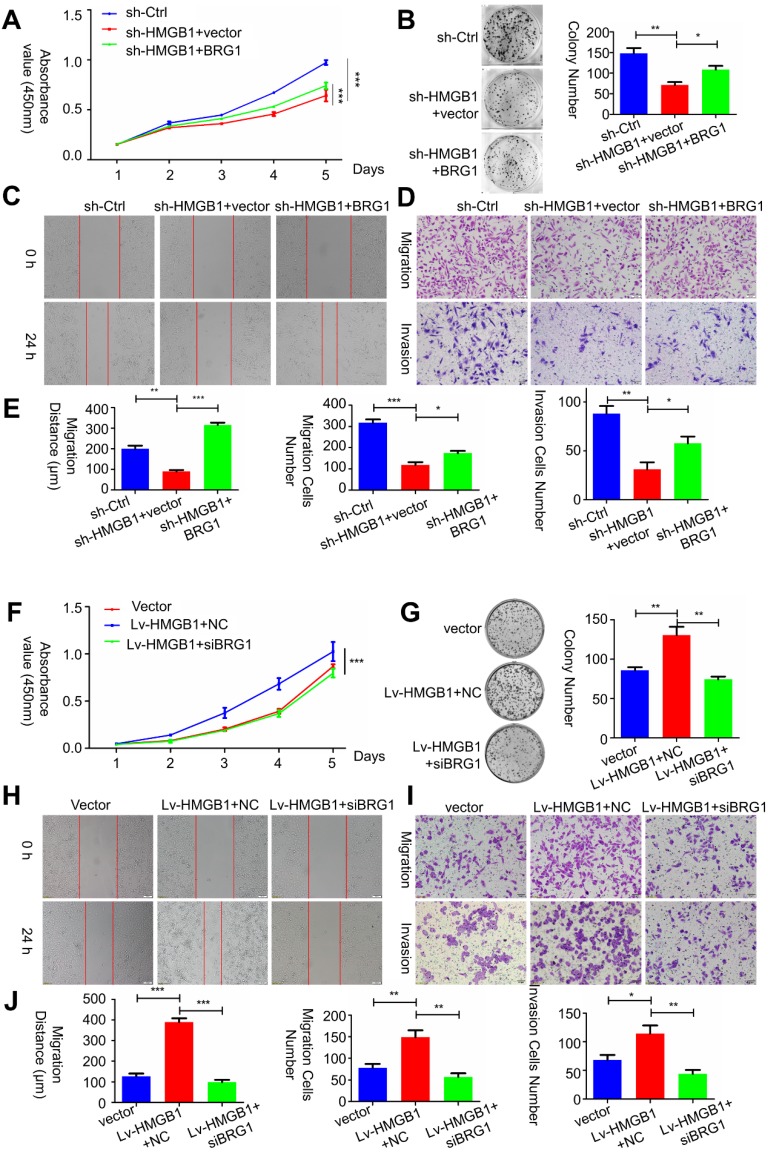
***HMGB1*****mediated carcinogenic effects in PCa cells via* BRG1.*** (**A-C**) Effects of co-transfection of *HMGB1*-shRNA and *BRG1* or *HMGB1* and si-*BRG1* on cell proliferation, invasion, and migration by CCK-8, colony formation, scratch-wound-healing, and Matrigel invasion assays. (**D** and** J**) Graphical illustration of statistical results of co-transfection of *HMGB1*-shRNA and *BRG1* or *HMGB1* and si-*BRG1* on cell migration and invasion. *P<0.05, **P<0.01, ***P<0.001, n = 3.

To determine whether *HMGB1*-mediated *BRG1* affected the cell viability as well as migration and invasion of PCa cells, co-transfection of *HMGB1* and si-*BRG1* or *HMGB1*-shRNA and *BRG1* was performed. Consist with EMT marker reversal, *BRG1* overexpression in *HMGB1*-knockdown cells impaired the migration and invasion of PC-3 cells (**Figure 6 A-E**). In contrast, depletion of *BRG1* remarkably impaired aggressive behavior of the *HMGB1*- overexpressing cells (**Figure 6F-J**). Moreover, Western blot analysis results demonstrated that blocking *Akt* activation excessively increased the expression of E-cadherin, but not vimentin (**Figure S4**), indicated that this aberrant signal triggered EMT process may partly contribute to the activation of *Akt* signaling pathway. Collectively, these results suggested that *HMGB1* mediates EMT of PCa cells via inducing *BRG1* expression.

### HMGB1 and BRG1 are co-expressed in PCa cells and PCa tumors

**Figure 7:**
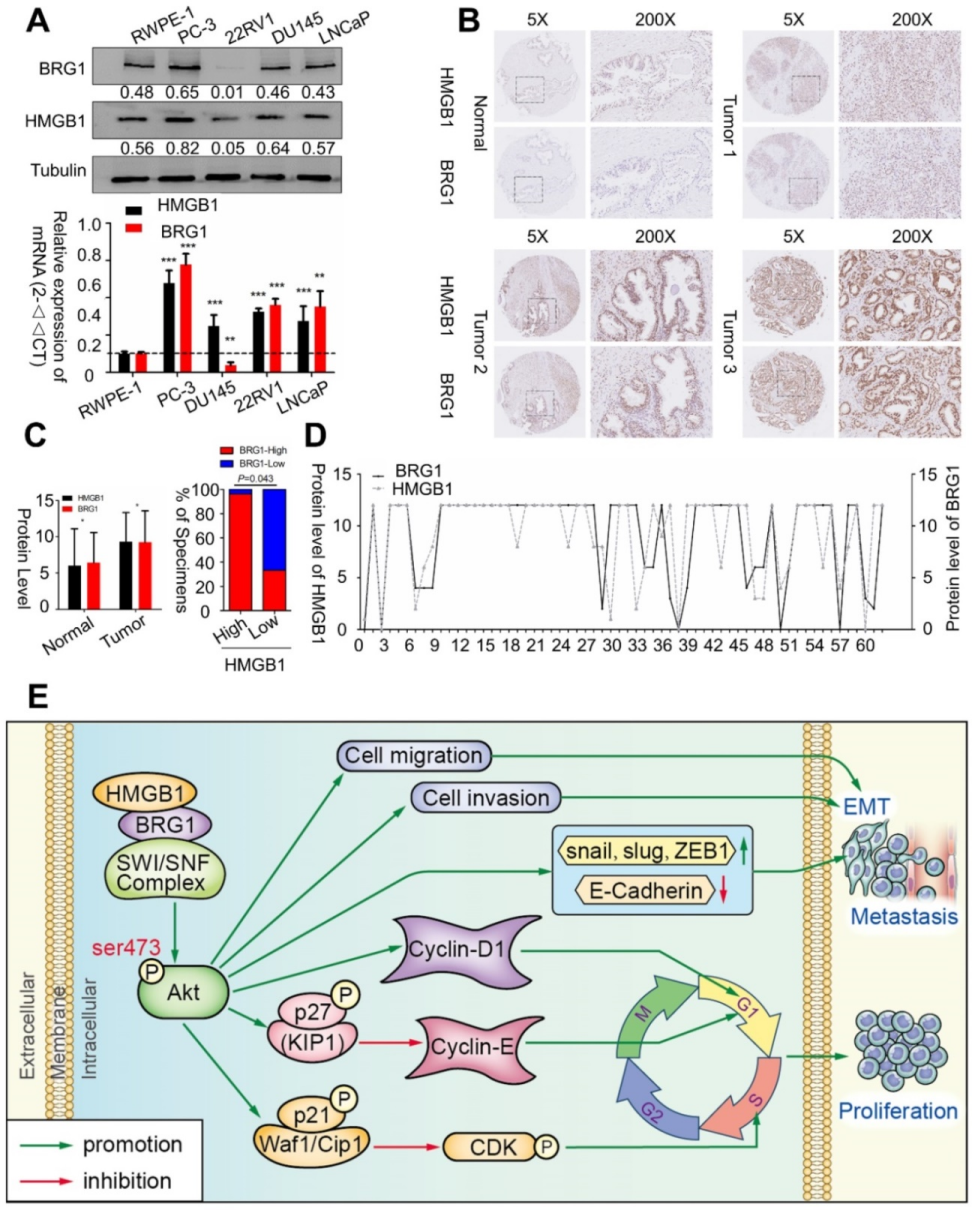
***BRG1* was positively correlated with *HMGB1* in PCa cells and tissues.** (**A**) *HMGB1* and *BRG1* protein and mRNA expression in the normal prostate epithelial cell line (RWPE-1) and five PCa cell lines were detected by Western blot (α-tubulin was used as loading control) and qRT-PCR. (**B**) *BRG1* and *HMGB1* expressions in 64 paraffin-embedded PCa tissue samples were detected by immunohistochemistry staining. *BRG1* and *HMGB1* were spatially correlated. *BRG1* and *HMGB1* expression levels in PCa tissue specimens were positively correlated. Four Representative immunohistochemical staining photographs of normal tissue (Normal) and tumor tissue samples (Tumor 1, Tumor 2 and Tumor 3) are shown as indicated. (**C**) Left panel: quantification of *BRG1* and *HMGB1* expression levels in PCa tumors (n = 64) which were significantly higher than the normal tissues (n = 14). Magnification: 200×. *P < 0.05 compared to normal tissue; Right panel: percentage of PCa specimens showing low or high *HMGB1* expression relative to the level of *BRG1*. (**D**) Spearman's correlation analysis showed a positive relationship between the *BRG1* and *HMGB1* levels in 62 human PCa tissues. r = 0.574, *P* < 0.001. (**E**) Schematic representation of the proposed mechanism of *HMGB1* in PCa cells.

To confirm whether *BRG1* is expressed in different PCa cell lines and in human PCa tissues, we evaluated *BRG1* and *HMGB1* proteins together with mRNA expression in control RWPE-1 cells and four different PCa cell lines by Western blotting and qRT-PCR. High expression of both *BRG1* and *HMGB1* was detected in PC-3, DU145, and LNCaP cells (**Figure 7A**). Furthermore, IHC of 64 paraffin- embedded human PCa tissues and 14 benign prostatic hyperplasia (BPH) or normal prostate samples showed that expression of both *HMGB1* (*P* = 0.008, **Table 1**) and *BRG1* (*P* = 0.045, **Table 1**) was dramatically increased in PCa tumors than the corresponding BPH tissues (**Figure 7B-C**). We then analyzed the correlation between the expression of these two proteins and clinicopathological variables. Chi‑square analysis demonstrated that expression of *HMGB1* and *BRG1* was significantly associated with tumor Gleason score/International Society of Urological Pathology (ISUP) Grade Group (*P_HMGB1_* = 0.01 and *P_BRG1_* = 0.006) and clinical stage (*P_HMGB1_* = 0.002 and *P_BRG1_* = 0.045), but not associated with patient's age, T stage, regional lymph nodes, or distant metastasis (**Table 2**). Furthermore, our results showed a spatial and protein level correlation between *HMGB1* and *BRG1* in prostate carcinoma tissues (r^2^ =0.574, *P*<0.001; **Figure 7D**) implying that *HMGB1* modulates *BRG1* function in prostate carcinoma progression.

## Discussion

Our previous study revealed a positive correlation between *HMGB1* and *RAGE* expression in a cohort of patients with primary prostate cancer 14 suggesting that *HMGB1* is implicated in the development and/or progression of PCa. To elucidate the pivotal role of increased expression of *HMGB1* in PCa, we performed loss-of-function and gain-of-function experiments. Our findings demonstrated that shRNA-mediated silencing of *HMGB1* expression in PC-3 and LNCaP cells significantly decreased cell proliferation, migration, and invasion and inhibited entry into the S-phase of the cell cycle. In the *in vivo* studies using a murine model, *HMGB1* silencing resulted in the suppression of tumorigenesis and lung metastasis. On the contrary, ectopic expression of *HMGB1* augmented the aggressive behavior of PCa cells. These observations underscored the key role of *HMGB1* in the proliferation and metastasis of PCa.

*HMGB1* is a chromatin-binding protein involved in DNA replication and DNA repair processes 25. Aberrant overexpression of *HMGB1* has been shown in a variety of cancers and is closely associated with tumorigenesis 6, 10. Previously, we have shown upregulation and co-expression of *RAGE* and *HMGB1* in PCa, which suggested a cooperative role of both proteins in the progression of PCa 14. Also, *HMGB1* may regulate *AR* either by acting as a co-activator of *AR* or indirectly associating with *RAGE* signaling in prostate oncogenesis 26. This implied that *HMGB1* may be involved in the tumorigenesis of PCa *via* multiple pathways. We, therefore, analyzed multiple key cell cycle proteins associated with the G_1_ to S phase transition by immunoblotting. There was a concomitant inhibition of *C-myc*, *CDK4*, *cyclin D1,* and *cyclin E* with the knockdown of *HMGB1*, whereas the expression of cyclin-dependent kinase inhibitors *p27* and *p21* was increased. *HMGB1* has also been shown to be involved in cell proliferation and oncogenesis by activating the *PI3K/Akt* pathway 12, 27. However, it was unclear whether *HMGB1* induced prostate carcinoma through *PI3K/Akt* pathway. We, therefore, analyzed the key proteins in this pathway and found that silencing of *HMGB1* resulted in remarkably decreased phosphorylated *Akt* levels, whereas elevated expression of *HMGB1* induced the phosphorylation of *Akt*. These results suggested that *HMGB1* played a critical role in PCa progression.

*HMGB1* has been implicated in the metastasis of different human malignancies including lung adenocarcinoma 28, triple-negative breast cancer 29, and osteosarcoma 30. Moreover, employing transgenic adenocarcinoma mouse prostate (TRAMP) model, He et al reported that *HMGB1* promotes invasive carcinoma 31. In the present study, we have shown that *HMGB1* is involved in the metastasis of PCa by regulating EMT in PCa cells. Tumor metastasis is attributed to many different mechanisms in various cancers; one of these mechanisms, EMT, is generally recognized to mediate the process of cancer cell invasion and metastasis 32. Our results demonstrated that knockdown of *HMGB1* resulted in elevated the expression of E-cadherin and reduced expression of *vimentin* which are considered characteristic features of EMT. However, the molecular mechanisms underlying *HMGB1*-mediated EMT in PCa remain unclear.

In the present study, using LC-MS/MS analysis, we defined *BRG1* from the candidate *HMGB1*- interacting proteins. *BRG1* encodes an ATPase subunit of the *SWI/SNF* chromatin- remodeling complex, which regulates transcriptional activity by remodeling the chromatin structure and is involved in signal transduction, gene transcription, and protein stability 33, 34. *BRG1* is highly conserved and located on chromosome 19p13.2, an area usually amplified n various tumors, including melanoma 35 and gastric 36 and PCa 37. Previous studies have shown that amplification of *BRG1* was highly correlated with metastatic phenotype and malignant progression 38. Notably, stabilization of *BRG1* suppressed *E-cadherin* expression in gastric cancer cells subsequently promoting metastasis 36. Furthermore, *BRG1* has been reported as a corepressor of *ZEB1* to regulate *E-cadherin* transcription and was required for the induction of EMT by *ZEB1* 24. It has been reported that activation of both *Akt* and other signaling pathways may induce EMT contributing to tumor metastasis 39. A recent study described that *BRG1* acted as a prognostic indicator and a potential therapeutic target for PCa 40. Increased *BRG1* expression in *PTEN*-deficient PCa cells led to chromatin remodeling into configurations that drove a pro-tumorigenic transcriptome, steering cells to become further addicted to *BRG1* 23.

Our data indicated that *HMGB1* acted as an interacting partner of *BRG1*. By increasing *BRG1* protein expression, *HMGB1* promoted the growth and invasion of PCa and mediated EMT. The correlation between *HMGB1* and *BRG1* was confirmed by the following observations. First, in PCa cell lines as well as in clinical specimens, the expression of *BRG1* significantly correlated with *HMGB1* levels. Second, the introduction of *HMGB1* dramatically increased the expression of BRG1 in PCa cells. Third, deletion of *BRG1* partly counteracted the aggressive phenotype mediated by *HMGB1*. And finally, ectopic expression of *BRG1* rescued the attenuation of cellular tumorigenesis and EMT by the knockdown of *HMGB1* indicating that *BRG1* was required for *HMGB1*-induced malignant progression in PCa. These results strongly demonstrate that BRG1 is a downstream effector of *HMGB1*.

Accumulating evidences have revealed that multiple cellular signaling pathways such as *Wnt/β‐catenin*, mitogen‐activated protein kinase (*MAPK*), *TGF‐β/Smads* were implicated in the progression of EMT 41-43. Evidence from recent literatures and our previous studies imply that *PI3K/Akt* signaling pathway palys a pivotal role in the EMT process of PCa 44, 45. It is has been widely reported that *PI3K/AKT* signaling pathway regulates EMT by promoting the phosphorylation of downstream target proteins (*Bad*, *Caspase9*, *NF‐κB*, *GSK‐3β*, *mTOR*, *p21Cip1*, *p27 Kip1*, etc.) to further mediates tumor proliferation, invasion and metastases 46-48. Furthermore, activation of* p‐Akt* could increase the expression of integrin‐linked kinase as well as induce key transcription factors, such as *Snail*, *Slug*, and *Twist*, which ultimately promotes the process of EMT 49-51. Both *HMGB1* and *BRG1* have been shown to be closely related with the *PI3K/AKT* pathway 12, 22. However, whether the activation of *Akt* contributes to the cellular metastasis function of PCa induced by *HMGB1-BRG1* axis remains uncertain. In the present study, we observed that *HMGB1* could activate *PI3K/AKT* signaling pathway by regulating the protein phosphorylation level and that *HMGB1*-induced activation of EMT occurs partly through phosphorylation of *Akt*. Furthermore, HMGB1 and BRG1 were required for increased cell migration and invasion of PCa cells by *Akt*. Meanwhile, blocking *Akt* activation with LY294002 largely increased the expression of E-cadherin, while showed no significant effect in vimentin inferred that *p‐Akt* protein levels fall below a certain threshold resulting in upregulation E-cadherin level and finally alleviated cell migration competency in PCa cells during EMT process.

No changes of vimentin protein expression implied E-cadherin was not interrelated to vimentin in PCa cells, which was consistent with previous reports 52, 53. Notably, loss of E-cadherin was reckoned as the crucial step to initiate EMT that sustained PCa metastasis 54. Based upon these observations, we propose that *HMGB1* may strengthen *BRG1* function and activate the *Akt* signaling pathway to promote EMT. In the future, it would be important to elucidate the mechanisms underlying the *HMGB1-*mediated upregulation of *BRG1* as well as the molecular interactions of *HMGB1* and *BRG1* implicated in EMT in PCa cells.

## Conclusions

In summary, our data indicated that *HMGB1* plays a vital role in tumorigenesis and metastasis of PCa process. *HMGB1* promotes PCa development *via* activation of the *Akt* signaling pathway and facilitates metastasis through modulating *BRG1*-mediated EMT (**Figure 7E**). *HMGB1* may serve as a molecular marker to monitor the progression of PCa.

## Supplementary Material

Supplementary figures and table legends.

Supplementary table 1.

Supplementary table 2.

## Figures and Tables

**Table 1:** Expression of *HMGB1* and *BRG1* in BPH and prostate cancer tissues.

		*HMGB1* protein expression		*P* value		*BRG1* protein expression		*P* value
Type	N	Positive (%)	Negative (%)		N	Positive (%)	Negative (%)	
** BPH**	14	8 (57.1)	6 (42.9)	0.008	14	10 (71.4)	4 (28.6)	0.045
** PCa**	62	56 (90.3)	6 (9.7)		64	60 (93.8)	4 (6.3)	

Values are n (%). A significantly increasing frequency of positive expression of *HMGB1* and *BRG1* was detected in prostate cancer specimens compared to BPH tissues (*P*=0.045, χ^2^-test)

**Table 2:** Correlation between *HMGB1* and *BRG1* expression with reference to clinicopathological characteristics was analyzed in prostate cancer (n=64)

Clinicopathological characteristics	N	*HMGB1*		*p* value^b^	N	*BRG1*		*p* value^b^
		Positive (%)	Negative (%)			Positive (%)	Negative (%)	
**Age(years)**								
≤ 69^a^	29	28 (96.6)	1 (3.4)	0.123	30	29 (96.7)	1 (3.3)	0.353
> 69	33	28 (84.8)	5 (15.2)		34	31 (91.2)	3 (8.8)	
**pT status**								
T1-T2	29	27 (93.1)	2 (6.9)	1.0	42	40 (95.2)	2 (4.8)	0.603
T3-T4	27	26 (96.3)	1 (3.7)		22	20 (90.9)	2 (9.1)	
**Clinical stage**								
I- II	15	10 (66.7)	5 (33.3)	**0.002**	16	13 (81.3)	3 (18.8)	**0.045**
III- IV	47	46 (97.9)	1 (2.1)		48	47 (97.9)	1 (2.1)	
**Gleason Score**								
< 7	7	4 (57.1)	3 (42.9)	**0.01**	8	5 (62.5)	3 (37.5)	**0.006**
≥ 7	51	49 (96.1)	2 (3.9)		52	51 (98.1)	1 (1.9)	
**N - Regional lymph nodes**								
N0	52	47 (90.4)	5 (9.6)	0.97	54	51 (94.4)	3 (5.6)	0.62
N1	10	9 (90.0)	1 (10.0)		10	9 (90.0)	1 (10.0)	
**M - Distant metastasis**								
M0	53	48 (90.6)	5 (9.4)	0.88	55	51(92.7)	4 (7.3)	0.26
M1	9	8 (88.9)	1 (11.1)		9	9 (100)	0 (0.0)	

HMGB1 and BRG1 expression was determined by IHC; a: mean age. b: *p*-value is from χ^2^-test -test.

## Abbreviations

*HMGB1*: high mobility group box 1
PCa: prostate cancer
*BRG1*: Brahma-related gene 1
EMT: epithelial-mesenchymal transition
FBS: fetal bovine serum
PCR: polymerase chain reaction
qRT-PCR: Quantitative reverse transcription polymerase chain reaction
RIPA: Radio-Immunoprecipitation Assay
BCA: bicinchonininic acid
SDS: dodecyl sulfate, sodium salt
TBS-T: triethanolamine buffered saline solution-Tween
ECL: enhanced chemiluminescence
PBS: Phosphate Buffered Saline
EDTA: Ethylene Diamine Tetraacetic Acid
NP40: NobleRyder 40
2 × SDS-PAGE: 2 × dodecyl sulfate, sodium salt-Polyacrylamide gel electrophoresis
DAPI: 4′, 6-Diamidino-2-phenylindole
