# Supplementary material for: HMGB1 Promotes Prostate Cancer Development and Metastasis by Interacting with Brahma-Related Gene 1 and Activating the Akt Signaling Pathway
Source: Theranostics. 2019 Jul 9;9(18):5166–82. doi: 10.7150/thno.33972 (PMC6691575; doi:10.7150/thno.33972)
Supplement: Supplementary file 1 — Supplementary figures and table legends. [file thnov09p5166s1.pdf]

HMGB1 Promotes Prostate Cancer Development and Metastasis by Interacting with Brahma-Related Gene 1 and Activating the Akt Signaling Pathway

Running title: *HMGB1* promotes cell growth and metastasis in PCa

Dao-Jun Lv[1], Xian-Lu Song[2], Bin Huang[1], Yu-Zhong Yu[1], Fang-Peng Shu[1], Chong Wang[1], Hong Chen[1], Hai-Bo Zhang[1], Shan-Chao Zhao[1*]

[1] Department of Urology, Nanfang Hospital, Southern Medical University/ The First School of Clinical Medicine, Southern Medical University, Guangzhou 510515, China;

[2] Department of Radiation Oncology, Affiliated Cancer Hospital and Institute of Guangzhou Medical University, Guangzhou 510095, China.

[*]Correspondence: Prof. SC Zhao (lv577531@i.smu.edu.cn)

Supplemental Data

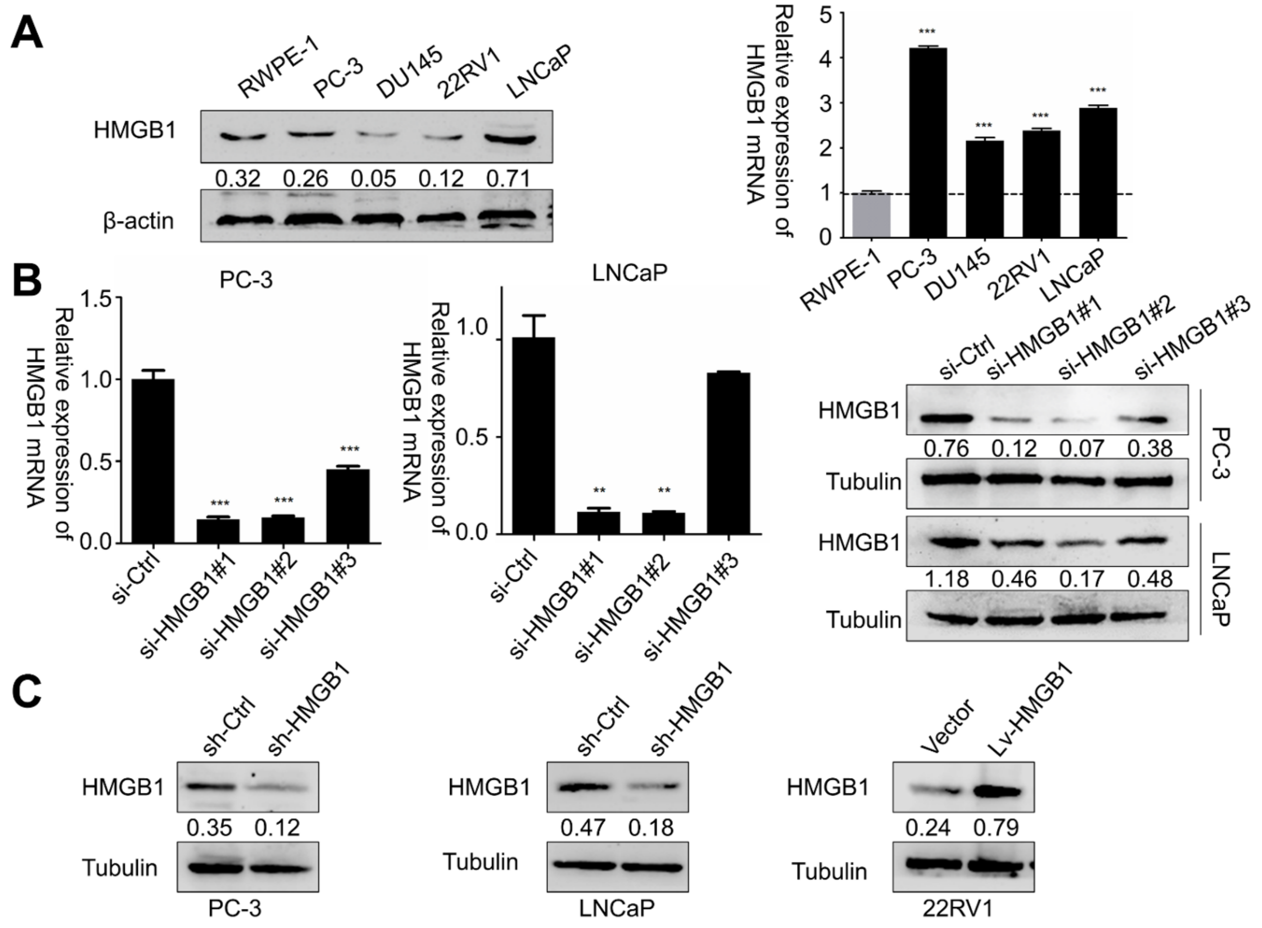

Supplemental Figure 1: *HMGB1* expression in human PCa cell lines and establishment of stable expression *HMGB1* cells. (**A**) Western blot and Real-time PCR analysis of *HMGB1* expression in PCa cell lines. (**B**) RNAi-silencing of *HMGB1* in shRNA-transduced stable PC-3 and LNCaP cells. (**C**) Western

blotting analyses of *HMGB1* in specific shRNA-transduced stable PC-3, LNCaP cells and 22Rv1 cell with ectopic expression of *HMGB1*. *α-Tubulin* was used as a loading control. *P<0.05, **P<0.01, ***P<0.001.

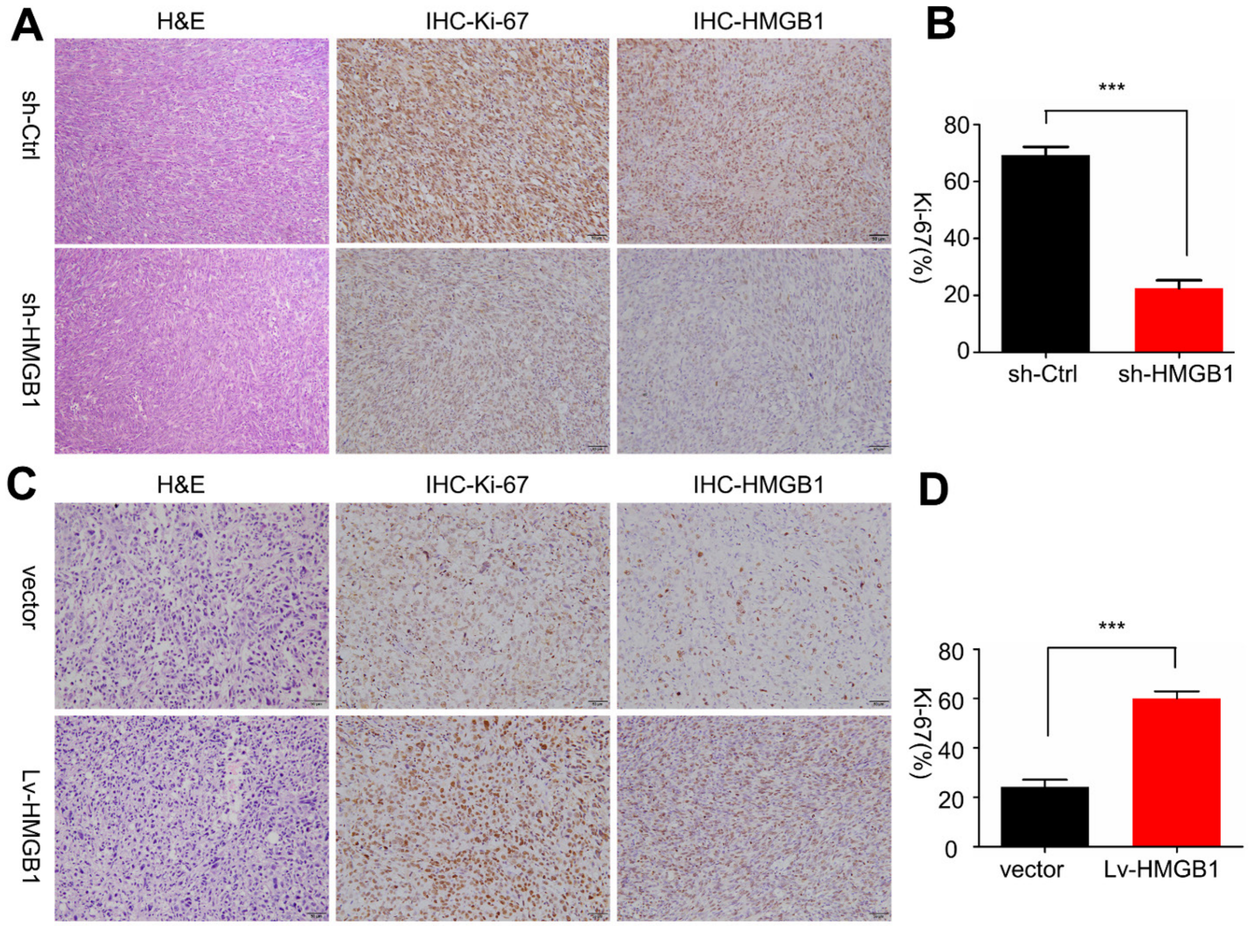

Supplemental Figure 2: *HMGB1* and *Ki-67* immunohistochemical staining of xenograft tumor tissues. (A, C) Representative photographs of tumor tissue structures (H&E) and *HMGB1* and *Ki-67* immunohistochemical staining of xenograft tumor tissues derived from control (sh-Ctrl) or silencing *HMGB1* (sh-*HMGB1*) shRNA-contained PC-3 (A) or overexpression of *HMGB1*(Lv-*HMGB1*) 22Rv1 cells (B) as indicated. Magnification: 200x. (B, D) Quantification of *HMGB1* and *Ki-67* expression. ***P<0.001 compared to the sh-Ctrl or empty vector group, n = 6.

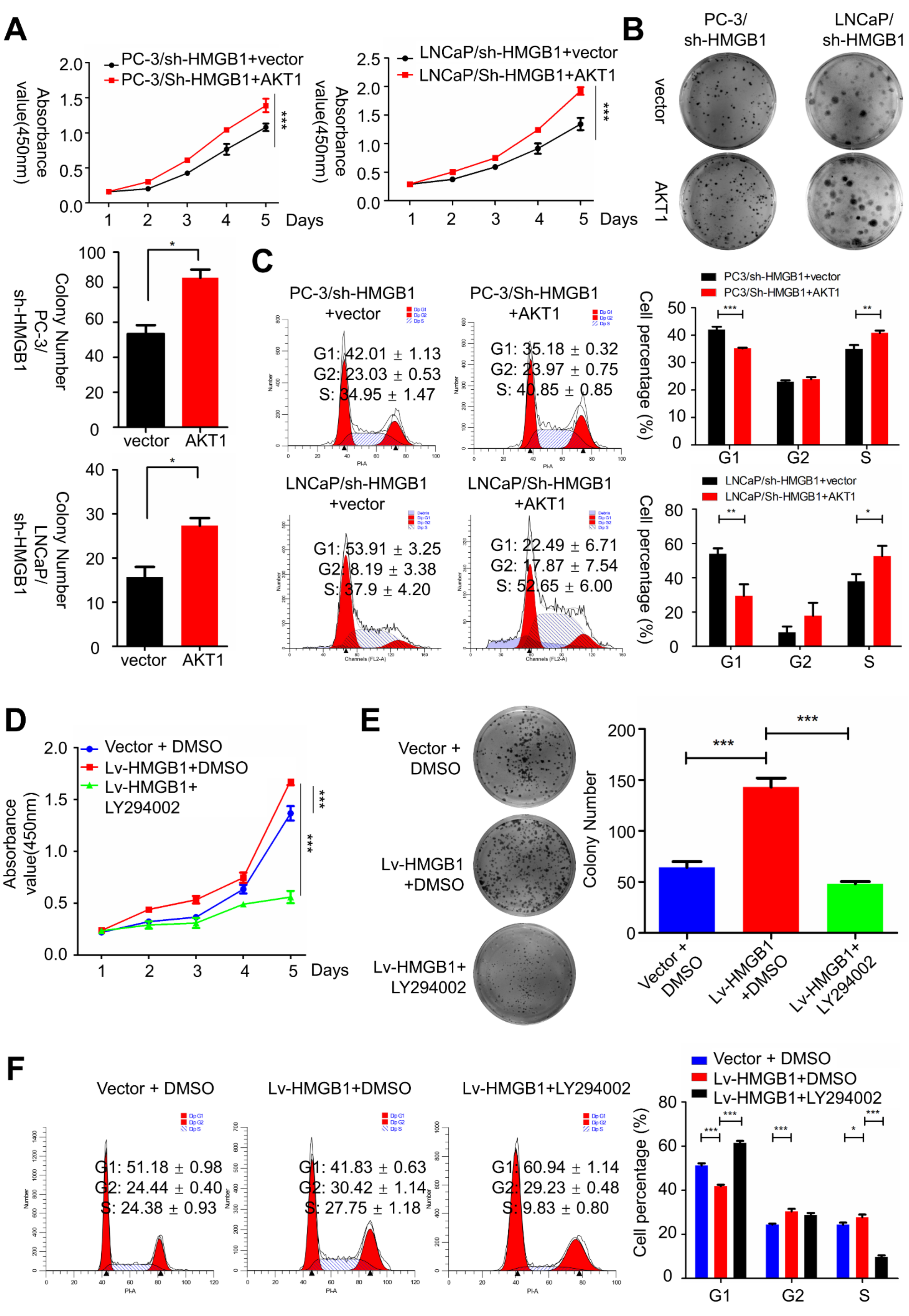

A
PC-3/sh-HMGB1+vector
PC-3/Sh-HMGB1+AKT1
Absorbance value(450nm)
Days
LNCaP/sh-HMGB1+vector
LNCaP/Sh-HMGB1+AKT1
B
PC-3/ sh-HMGB1
LNCaP/ sh-HMGB1
vector
AKT1
Colony Number PC-3/ sh-HMGB1
Colony Number LNCaP/ sh-HMGB1
vector
AKT1
C
PC-3/sh-HMGB1 +vector
G1: 42.01 ± 1.13
G2: 23.03 ± 0.53
S: 34.95 ± 1.47
PC-3/Sh-HMGB1 +AKT1
G1: 35.18 ± 0.32
G2: 23.97 ± 0.75
S: 40.85 ± 0.85
PC3/sh-HMGB1+vector
PC3/Sh-HMGB1+AKT1
Cell percentage (%)
LNCaP/sh-HMGB1 +vector
G1: 53.91 ± 3.25
G2: 8.19 ± 3.38
S: 37.9 ± 4.20
LNCaP/Sh-HMGB1 +AKT1
G1: 22.49 ± 6.71
G2: 17.87 ± 7.54
S: 52.65 ± 6.00
LNCaP/sh-HMGB1+vector
LNCaP/Sh-HMGB1+AKT1
G1
G2
S
D
Vector + DMSO
Lv-HMGB1+DMSO
Lv-HMGB1+ LY294002
Absorbance value(450nm)
Days
E
Vector + DMSO
Lv-HMGB1 +DMSO
Lv-HMGB1+ LY294002
Colony Number
F
Vector + DMSO
G1: 51.18 ± 0.98
G2: 24.44 ± 0.40
S: 24.38 ± 0.93
Lv-HMGB1+DMSO
G1: 41.83 ± 0.63
G2: 30.42 ± 1.14
S: 27.75 ± 1.18
Lv-HMGB1+LY294002
G1: 60.94 ± 1.14
G2: 29.23 ± 0.48
S: 9.83 ± 0.80
Cell percentage (%)
Vector + DMSO
Lv-HMGB1+DMSO
Lv-HMGB1+LY294002

Supplemental Figure 3: *HMGB1* activates *Akt* signaling pathways. (**A-B**) Transfection of constitutively activated *Akt* (myr-*Akt*) into PC3-sh*HMGB1* and LNCaP-sh*HMGB1* cells restored proliferation, as determined by CCK-8 assays and colony formation assays. (**C**) Upon myr-*Akt* transfection into PC3-*HMGB1*-shRNA and LNCaP-*HMGB1*-shRNA cells, the percentage of cells in G_1 phase decreased and the percentage in S phase increased. (**D, E and F**) Inhibition of the *Akt* signaling blocks the promoting effect of *HMGB1*-overexpression on cell proliferation and cell cycle progression of PCa cells as determined by CCK-8 assay (**D**), colony formation assay (**E**) and flow cytometry (**F**) after treatment with LY294002 (50 μM). Error bars represent mean ± SD from 3 independent experiments; *P<0.05, **P<0.01 and ***P<0.001.

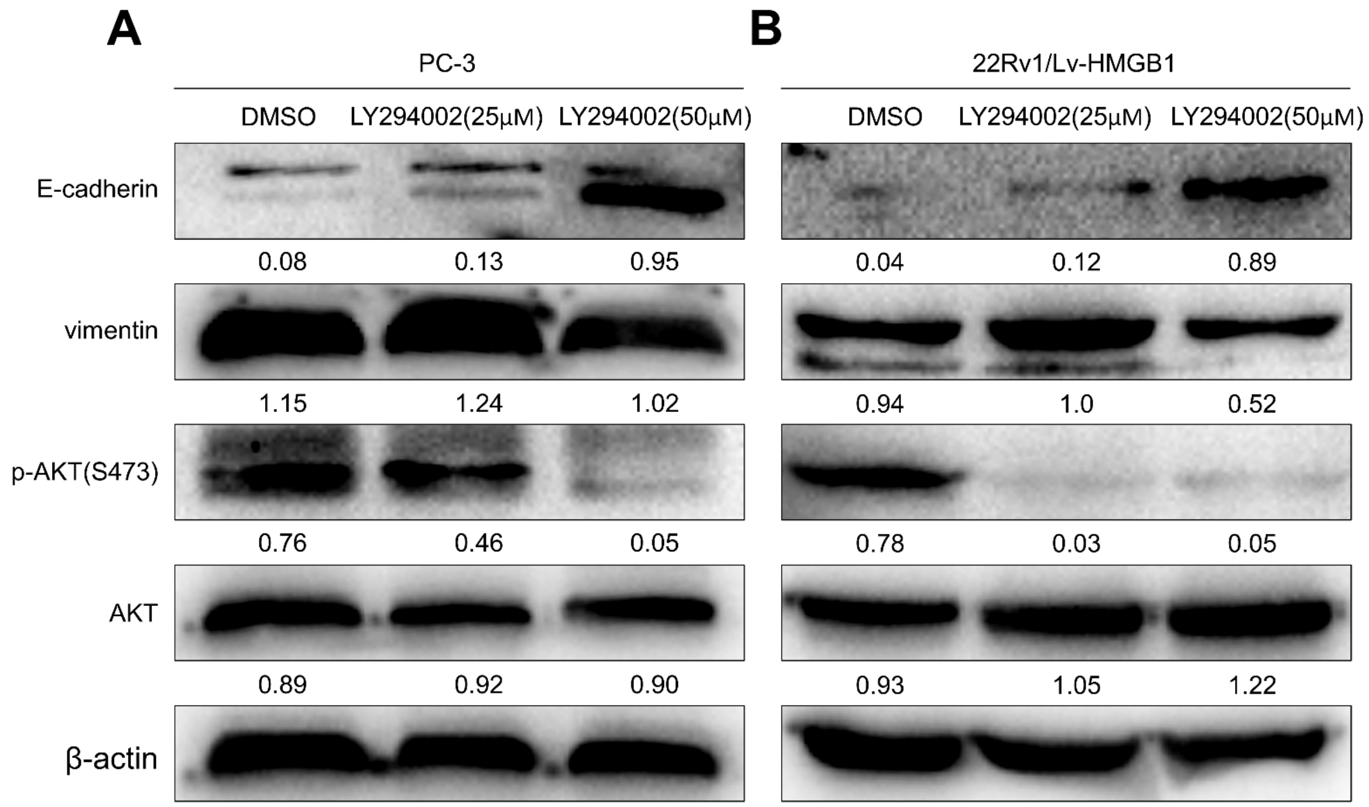

Supplemental Figure 4: LY294002 blocked *Akt* phosphorylation in PC-3 and 22Rv1/Lv-*HMGB1* cells. Western blot analysis was performed to evaluate p - *Akt*, *Akt* and EMT markers expression under LY294002 treatment for 48 h in PC-3(**A**) and 22Rv1/Lv-*HMGB1*(**B**). p - Akt: phosphor - *Akt*.

Supplemental Table1: The information of proteins analyzed from LC-MS-MS.

Supplemental Table2: The characteristics of patients and the stain score of *HMGB1* and *BRG1*.
